# The Influence of the Coaches’ Demographics on Young Swimmers’ Performance and Technical Determinants

**DOI:** 10.3389/fpsyg.2020.01968

**Published:** 2020-08-11

**Authors:** Daniel A. Marinho, Tiago M. Barbosa, Vitor P. Lopes, Pedro Forte, Argyris G. Toubekis, Jorge E. Morais

**Affiliations:** ^1^Department of Sports Sciences, University of Beira Interior, Covilhã, Portugal; ^2^Research Center in Sports, Health and Human Development (CIDESD), University of Beira Interior, Covilhã, Portugal; ^3^Department of Sports Sciences, Instituto Politécnico de Bragança, Bragança, Portugal; ^4^Department of Sports Sciences, Douro Higher Institute of Educational Sciences, Penafiel, Portugal; ^5^Sports Performance Laboratory, School of Physical Education and Sport Science, National and Kapodistrian University of Athens, Athens, Greece

**Keywords:** biomechanics, efficiency, talent identification, youth, swimming

## Abstract

The purpose of this study was to understand the relationship between the coaches’ demographics (academic degree and/or coaching level and/or coaching experience) and young swimmers’ performance and technical ability. The sample was composed by 151 young swimmers (75 boys and 76 girls: 13.02 ± 1.19 years old, 49.97 ± 8.77 kg of body mass, 1.60 ± 0.08 m of height, 1.66 ± 0.09 m of arm span), from seven different clubs. Seven coaches (one per club) were responsible for the training monitoring. Performance and a set of biomechanical variables related to swim technique and efficiency were assessed. The swimmers’ performance was enhanced according to the increase in the coaches’ academic degree (1: 75.51 ± 10.02 s; 2: 74.55 ± 9.56 s; 3: 73.62 ± 7.64 s), coaching level (1: 76.79 ± 11.27 s; 2: 75.06 ± 9.31 s; 3: 73.65 ± 8.43 s), and training experience (≤5-y training experience: 75.44 ± 9.57 s; >5-y training experience: 74.60 ± 9.54 s). Hierarchical linear modeling retained all coaches’ demographics characteristics as main predictors (being the academic degree the highest: estimate = -1.51, 95% confidence interval = -0.94 to -2.08, *p* = 0.014). Hence, it seems that an increase in the demographics of the coaches appears to provide them with a training perspective more directed to the efficiency of swimming. This also led to a higher performance enhancement.

## Introduction

In youth sports, training and performance are the main topics of interest for researchers and practitioners (Morais et al., 2016). However, less attention is given to the influence that coaches may induce in their athletes. They are the ones bridging researchers and support staff (producing evidence-based knowledge and recommendations) and the athletes (the end users of such knowledge and services) (Crowcroft et al., 2020).

Coaches are perceived by both athletes and their significant others (i.e., parents) as role models and key players in making sports experience beneficial for children, as well as facilitating the athletes’ social and motor development (Fry and Gano-Overway, 2010). Moreover, it was pointed out that a task-orientation environment facilitated by a coach is highly helpful to the commitment toward the sports activity in youth (Martinent and Decret, 2015). However, this begs the question if coaches with a variety of demographics, backgrounds and experiences will be able to empower athletes to engage in effective task-oriented activities and ultimately to achieve success.

Over the long-term development, a mutual dependence is verified based on the athletes’ need to acquire knowledge, competence, and experience from the coach, whereas the coaches need to promote performance and success of the athletes based on their own competences and skills (Philippe and Seiler, 2006). A study by Crowcroft et al. (2020) showed that the use of athlete-monitoring tools improved the coaches’ prediction to identify performance changes in adult swimmers. However, young athletes do not display a learning or knowledge acquisition pattern like their older counterparts. One can argue that the perception that athletes may have depends on the type of communication used by the coach to convey the message, which can be related to their background (i.e., demographics) (Santi et al., 2014). Indeed, It was argued that an effective coach is seen as being proficient whenever he/she sets targets, or establishes priorities of intervention (Grosso, 2006). Moreover, it was suggested that these professionals need to begin thinking differently not only about how they coach but also about the nature and truth of the knowledge that informs their coaching, including pedagogical and technical content (Denison, 2010; Gilbert and Côté, 2013). Therefore, it can be suggested that coaches with different demographics may denote or use different communication skills and adopt different coaching styles based on the knowledge they acquire over time. To the best of our knowledge, literature does not report yet how the coaches’ demographics (e.g., academic training, coaching training, coaching background) can contribute to young swimmers’ performance.

Additionally, age-group coaches face the challenge of designing a development program that must feature an effective dose-response. For one side, there is the need to elicit a performance improvement over time (Yustres et al., 2020); on the other, tackling concerns on side effects of heavy and demanding programs, such as musculoskeletal injuries or psychological issues (e.g., burnout) (Monteiro et al., 2018). A well-designed training program should provide appropriate stimulation in order to produce the expected adaptations (Barroso et al., 2015; Whitworth-Turner et al., 2019). In swimming, some concerns have been raised about youth swimming training workload and periodization (Lang and Light, 2010). Literature suggests two training approaches to be employed in age-group swimming: (1) focusing on high training volume (quantity), or alternatively, (2) on training efficiency (quality) (Nugent et al., 2017). On one side of the debate, the argument is that programs based on large mileage would promote sharp improvements in aerobic fitness, but will not make the swimmers faster (Salo and Riewald, 2008). On the other side, literature reports that young swimmers’ performance is highly determined by technical factors (i.e., related to swim efficiency) (Morais et al., 2017). Nonetheless, this approach is seen as a long-term development, and as such, it may not yield short-term effects.

A growing body of knowledge has been reporting that the performance enhancement should be focused on the development and consolidation of the technique, based on a long-term development approach (Morais et al., 2017). The determinant factors related to stroke mechanics (i.e., technique) are the best predictors of young swimmers’ performance (by 60–85%) (Morais et al., 2012; Zacca et al., 2020). Therefore, it can be argued that the coaches’ demographics might facilitate better performances of swimmers under them, based on the enhancement of efficiency variables rather than large mileages. Nevertheless, a definitive answer to this remains elusive until now. Even though there were some educated guesses by practitioners, we have failed to find evidence-based knowledge that could be aiding an informed decision-making.

Therefore, the main purpose of this study was to understand the relationship between the coaches’ demographics (academic degree, coaching level, training experience) in the applied training content and the swimmers’ technical ability and performance. It was hypothesized that the existence of significant differences in the coaches’ demographics and training experience would have a meaningful contribution to the young swimmers’ performance and its determinants; i.e., higher level of education and longer career experience by coaches will be related to better efficiency by the swimmers.

## Materials and Methods

### Swimmers

The sample comprised 151 (75 boys and 76 girls) young swimmers (13.02 ± 1.19 years old, 49.97 ± 8.77 kg of body mass, 1.60 ± 0.08 m of height, 1.66 ± 0.09 m of arm span) with 3.36 ± 0.77 years of training experience. These were enrolled in a talent identification program, including national record holders, and swimmers participating regularly in regional and national events. Swimmers were assessed at the end of the second macrocycle of a competitive season (winter’s peak competition). Parents or guardians and the swimmers themselves signed an informed consent form. All procedures were in accordance to the Declaration of Helsinki regarding human research, and the university ethics board approved the research design.

### Coaches

The coaches’ sample was recruited from seven different swim clubs. Coaches did not provide information about the training workload, but all of them followed the same guidelines on young swimmers’ training periodization (Chatard and Stewart, 2011). Seven coaches (six males and one female: 31.52 ± 4.01 years old, with 7.29 ± 3.30 years of training experience), one per swim club, were responsible for designing and monitoring the training program. All of them held a coaching level certification (level 1: two coaches, level 2: three coaches, level 3: two coaches). Four held concurrently a bachelor’s degree, two a master degree in science, and one a philosophy doctor’s degree. The sample was split up according to their academic degree: graduated, 1; master in science, 2; philosophy doctor, 3. Coaching level: level 1, 1; level 2, 2; level 3, 3. Coaching experience: equal to or less than 5 years of coaching experience, ≤5 years; more than 5 years of coaching experience, >5 years.

### Research Design

This was a cross-sectional study aiming to understand the relationship between coaches’ demographics and swimmers’ performance and technical determinants. Besides the 100 m freestyle event, a comprehensive set of biomechanical variables was selected to be assessed. As aforementioned, such variables account for 60–85% of young swimmers’ performance at front-crawl stroke, enhancing their importance in youth swimming (Morais et al., 2012; Zacca et al., 2020). Moreover, there is a set of biomechanical variables that are strongly related to swim efficiency and hence deemed as efficiency proxies (Barbosa et al., 2010, 2015): (1) the stroke length (SL) is the distance swam per stroke cycle (Craig and Pendergast, 1979); (2) the stroke index (SI) describes the ability of swimming at a given velocity with the fewest number of strokes possible (Costill et al., 1985); (3) the Froude efficiency (η_F_) is the ratio between the useful mechanical work and the total mechanical work; i.e., it is the efficiency at which the thrust is converted into “useful” work (the work to overcome drag force) (Zamparo et al., 2020); and (4) the intracyclic variation of the swim velocity (*dv*) is the balance of instantaneous thrust (acceleration) and drag (deceleration) (Barbosa et al., 2010). Additionally, young swimmers’ technical training is also focused on hydrodynamic resistance, as better performances are related to less water resistance (Morais et al., 2012; Barbosa et al., 2019). Hence, a set of hydrodynamic variables was assessed: (1) the Froude number (*F*_r_) is a dimensionless variable that is deemed as a wave-making resistance index; (2) the active drag coefficient (*C*_Da_) quantifies the resistance of a swimmer displacing in water; and (3) the Reynolds number (*R*_e_) quantifies the water flow status around the swimmer (i.e., the level of turbulence that a swimmer creates displacing through water) (Kjendlie and Stallman, 2008).

### Performance

The 100 m freestyle event (short course meter, i.e., 25 m swimming pool) was selected as performance outcome. The time gap between the data collection and the 100 m freestyle event was no longer than 15 days as reported elsewhere (Morais et al., 2012).

### Kinematics and Efficiency

Swimmers were invited to undergo three maximal trials of 25 m at front-crawl and push-off start (at least 30 min of rest). A speedometer string (Swim Speedo-meter; Swimsportec, Hildesheim, Germany) was attached to the swimmers’ hip. An in-house built software (LabVIEW, v. 2010) was used to acquire (*f* = 50 Hz) and display speed-time data over each trial (Barbosa et al., 2016). Data was transferred by a 12-bit resolution acquisition card (USB-6008; National Instruments, Austin, TX, United States). Afterward, it was imported into a signal processing software (AcqKnowledge v. 3.9.0; Biopac Systems, Santa Barbara, CA, United States). Signal was handled with Butterworth fourth-order low-pass filter (cutoff: 5 Hz). Swim velocity (v, in m⋅s^–1^) over the trial was measured between the 11th- and 24th-meter mark. The stroke frequency (SF, in Hz) was calculated by the number of cycles per unit of time, from the time it takes to complete one full cycle (*f* = 1/*P*, where *P* is the period), and afterward converted to Hz. The mean of three consecutive full stroke cycles was afterward used for analysis.

The SL was calculated as SL = *v*/SF, where SL is the SL (m), *v* the swim velocity (m⋅s^–1^), and SF the stroke frequency (Hz) (Craig and Pendergast, 1979). The *dv* was computed as follows:

(1) $$d\,v=\frac{\sqrt{\frac{\sum_{i}\left(v_{i}-\bar{v}\right)\cdot F_{i}}{n}}}{\frac{\sum_{i}v_{i}\cdot F_{i}}{n}}\cdot 100$$

where *dv* is the intracyclic variation of the swim velocity (%), *v* is the mean swimming velocity (m⋅s^–1^), *v*_i_ is the instant swimming velocity (m⋅s^–1^), *F*_i_ is the acquisition frequency, and *n* is the number of observations (Barbosa et al., 2010). The SI was computed as follows: SI = *v*⋅SL, where SI is the SI (m^2^⋅s^–1^), *v* the swim velocity (m⋅s^–1^), and SL the SL (m) (Costill et al., 1985). The η_F_ was calculated as follows:

(2) $$\eta_{\text{F}}=\left(\frac{v\cdot 0.9}{2\,\pi \cdot S\,F\cdot l}\right)\cdot \frac{2}{\pi }\cdot 100$$

where η_F_ is the Froude efficiency (%), *v* the swim velocity (m⋅s^–1^), SF the stroke frequency (Hz), and *l* the shoulder to hand average distance (m) (Zamparo et al., 2020). The *l* was measured between the acromion and tip of the third finger, on dry land, whereas the swimmer was simulating a stroke cycle by digital photogrammetry (Morais et al., 2012).

### Hydrodynamics

The active drag coefficient (*C*_Da_, dimensionless) was computed with the velocity perturbation method (Kolmogorov and Duplishcheva, 1992). Swimmers were invited to perform two maximal trials at front crawl: one trial towing a hydrodynamic body (perturbation device) and the other without (Kolmogorov and Duplishcheva, 1992). The swim velocity was calculated as follows: *v* = *d*/*t*. The active drag (*D*_a_, in *N*) was computed as follows:

(3) $$D_{a}=\frac{D_{b}\,v_{b}\,v^{2}}{v^{3}-v_{b}^{3}}$$

where *D*_a_ is the swimmers’ active drag at maximal velocity (*N*); *D*_b_ is the resistance of the hydrodynamic body computed from the manufacturer’s calibration of the buoy-drag characteristics and its velocity (*N*); and *v*_b_ and *v* are the swim velocities with and without the perturbation device (m⋅s^–1^). Afterward, the *C*_Da_ was computed as follows:

(4) $$C_{D\,a}=\frac{2\cdot D_{a}}{\rho \cdot T\,T\,S\,A\cdot v^{2}}$$

where *C*_Da_ is the active drag coefficient (dimensionless); *D*_a_ is the active drag (*N*); ρ is the density of the water (being 1,000 kg⋅m^–3^); TTSA is the trunk transverse surface area (m^2^); and *v* the swim velocity (m⋅s^–1^).

The TTSA (in cm^2^) was measured by digital photogrammetry (Morais et al., 2012). The swimmers were invited to put their arms fully extended above the head, one hand over the other; fingers also extended close together and head in neutral position. They were photographed by a digital camera (Alpha 6000; Sony, Tokyo, Japan) in the transverse plane (downward view) on land simulating such streamlined position. Afterward, the TTSA was measured in a specific software (Udruler; AVPSoft, United States) (intraclass correlation coefficient = 0.987).

The Froude number (*F*_r_, dimensionless) was computed as follows:

(5) $$F_{r}=\frac{v}{\sqrt{g\cdot H}}$$

where *F*_r_ is the Froude number (dimensionless); *v* is the swim velocity (m⋅s^–1^), g is the gravitational acceleration (9.81 m⋅s^–2^); and *H* is the swimmer’s height (m) (Kjendlie and Stallman, 2008). The Reynolds number (*R*_e_, × 10^6^) was computed as follows:

(6) $$R_{e}=\frac{v\cdot H}{\upsilon }$$

where *R*_e_ is the Reynolds number (dimensionless); *v* is the swim velocity (m⋅s^–1^); *H* is the height (m); and υ is the water kinematic viscosity (being 8.97 × 10^–7^ m^2^⋅s^–1^ at 26°C) (Kjendlie and Stallman, 2008).

### Statistical Analysis

The Kolmogorov and Levene tests were applied to check the normality and homoscedasticity assumptions, respectively. Mean + 1 standard deviation was computed as descriptive statistics. The relative difference (Δ, in %) was calculated to verify the magnitude of the difference between each group of swimmers (i.e., comparison between groups of each coach’s demographic characteristics). One-way analysis of variance (ANOVA) (*p* < 0.05) was selected to verify the variation (coach effect) between each coach’s demographics (i.e., academic degree and coaching level). Afterward, Bonferroni test (*p* < 0.05) was used to verify differences between pairwise. The independent-sample *t*-test (*p* < 0.05) was selected to compare coaching experience (only two groups). The total η^2^ was selected as effect size index of the ANOVAs and deemed as follows: (1) without effect if 0 < η^2^ ≤ 0.04; (2) minimum if 0.04 < η^2^ ≤ 0.25; (3) moderate if 0.25 < η^2^ ≤ 0.64; and (4) strong if η^2^ > 0.64 (Ferguson, 2009). Cohen *d* was selected as a standardized effect size between pairwise comparisons and deemed as (1) small effect size 0 ≤ |*d*| ≤ 0.2, (2) medium effect size if 0.2 < |*d*| ≤ 0.5, and (3) large effect size if |*d*| > 0.5 (Cohen, 1988).

The relationship of the coaches’ demographics with the performance and technical determinants was computed by hierarchical linear modeling (HLM). This statistical procedure creates a hierarchical structure (i.e., a “tree”), being able to identify the independent variables (coaches’ demographics) as performance and technical determinants changing predictors. Two levels were used: (1) the first level included the swimmers’ sex; and (2) the second level included the coaches’ information (i.e., academic degree, coaching level, and coaching experience). The final model included only significant predictors. Maximum likelihood estimation was calculated with HLM7 software (Raudenbush et al., 2011).

## Results

Table 1 presents the ANOVA (i.e., coach effect) on the coaches’ academic degree and coaching level they were holding, as well as the *t*-test comparison between coaching experiences. It also presents the relative difference between groups, on each coach’s demographics.

**TABLE 1:** One-way ANOVA and *t*-test (coaching experience: two groups), relative difference (Δ, in%) and Cohen *d* (effect size) between groups, according to coaches’ demographics (academic degree, coaching level, and coaching experience).

	Academic degree					Coaching level					Coaching experience	
			Δ (*d*)					Δ (*d*)			Δ (*d*)	
	*F* ratio (*p*)	η^2^	1 vs. 2	2 vs. 3	1 vs. 3	*F* ratio (*p*)	η^2^	1 vs. 2	2 vs. 3	1 vs. 3	*t*-test (*p*)	≤5 y vs. >5 y
Performance (s)	0.38 (0.683)	0.006	1.29 (0.10)	1.26 (0.11)	2.57 (0.21)	1.11 (0.333)	0.016	2.30 (0.17)	1.90 (0.16)	4.25 (0.31)	0.52 (0.607)	1.13 (0.09)
*v* (m⋅s^–1^)	0.59 (0.554)	0.008	1.53 (0.13)	1.50 (0.13)	3.01 (0.26)	1.30 (0.275)	0.018	3.08 (0.25)	1.52 (0.13)	4.55 (0.35)	0.81 (0.419)	1.53 (0.19)
SL (m)	2.19 (0.116)	0.032	1.94 (0.19)	3.73 (0.31)	5.59 (0.47)	1.32 (0.272)	0.019	1.95 (0.19)	1.91 (0.17)	3.82 (0.35)	0.58 (0.564)	1.29 (0.12)
SI (m^2^⋅s^–1^)	0.79 (0.457)	0.011	1.49 (0.08)	4.27 (0.19)	5.69 (0.26)	1.30 (0.275)	0.019	6.37 (0.34)	0.49 (0.02)	6.83 (0.32)	0.40 (0.688)	1.48 (0.07)
η_F_ (%)	3.03 (0.052)	0.042	6.67 (0.57)	0.00 (0.00)	6.67 (0.57)	3.25 (0.042)	0.045	3.45 (0.25)	3.33 (0.28)	6.67 (0.57)	0.57 (0.572)	0.00 (0.00)
*dv* (%)	0.26 (0.776)	0.004	1.87 (0.06)	3.30 (0.15)	5.23 (0.17)	0.95 (0.390)	0.014	7.76 (0.22)	1.46 (0.05)	9.34 (0.29)	2.73 (0.007)	14.88 (0.45)
*F*_r_ (dimensionless)	0.40 (0.672)	0.006	3.03 (0.33)	0.00 (0.00)	3.03 (0.33)	1.75 (0.178)	0.025	3.03 (0.28)	0.00 (0.00)	3.03 (0.28)	0.97 (0.335)	3.03 (0.33)
*C*_Da_ (dimensionless)	6.16 (0.003)	0.123	31.58 (0.69)	5.56 (0.17)	38.89 (0.84)	9.76 (< 0.001)	0.181	28.30 (0.92)	43.24 (0.93)	2.70 (0.09)	1.05 (0.296)	12.20 (0.22)
*R*_e_ (× 10^6^ ± × 10^5^)	0.13 (0.882)	0.002	1.30 (0.09)	0.43 (0.03)	0.87 (0.06)	0.12 (0.890)	0.002	0.00 (0.00)	1.30 (0.09)	1.30 (0.08)	0.50 (0.619)	1.30 (0.09)

*Performance, 100 m freestyle event; v, swim velocity; SL, stroke length; SI, stroke index; η_*F*_, Froude efficiency; dv, intracyclic variation of the swimmer’s hip; F_*r*_, Froude number; C_*Da*_, active drag coefficient; R_*e*_, Reynolds number. Δ, relative difference (%); d, Cohen d. Academic degree: 1, bachelor degree; 2, master in science; 3, philosophy doctor. Coaching level: 1, level 1; 2, level 2; 3, level 3. Coaching experience: ≤5 y, equal and/or less 5 years of training experience; >5 y, over 5 years of training experience.*

**FIGURE 1:**
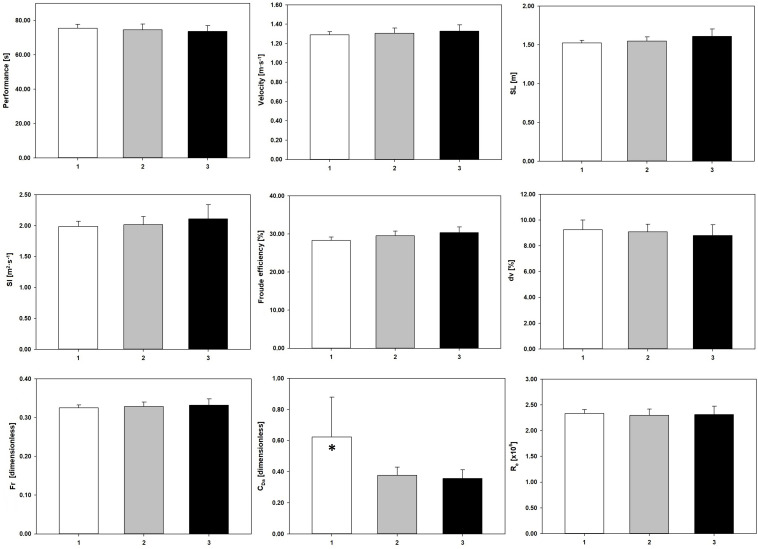
Performance and efficiency variables clustered by the coaches’ academic degree. Performance, 100 m freestyle performance; velocity, swim velocity; SL, stroke length; SI, stroke index; η_F_, Froude efficiency; *dv*, intracyclic variation of the swimmer’s velocity; *F*_r_, Froude number; *C*_Da_, active drag coefficient; *R*_e_, Reynolds number. 1, bachelor degree; 2, master in science; 3, philosophy doctor. ^∗^Significant differences (*p* < 0.05) between: 1 vs. 2, and 1 vs. 3.

Figure 1 depicts the variables assessed, clustered by the academic degree coaches were holding. The swimmers’ performance had an improvement (i.e., swam faster) with an increase in the coaches’ academic background (1: 75.51 ± 10.02 s; 2: 74.55 ± 9.56 s; 3: 73.62 ± 7.64 s), but without a significant variation (Table 1). The *C*_Da_ presented a significant variation, with significant differences between groups 1 and 2 (*p* = 0.015, Δ = 31.58%, *d* = 0.69), and between groups 1 and 3 (*p* = 0.017, Δ = 38.89%, *d* = 0.84) (Table 1).

**FIGURE 2:**
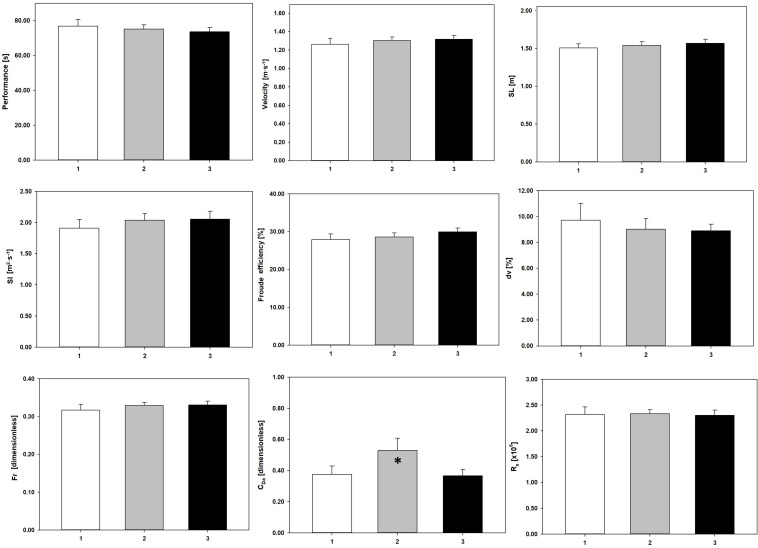
Performance and efficiency variables clustered by the coaches’ coaching level. Performance, 100 m freestyle performance; velocity, swim velocity; SL, stroke length; SI, stroke index; η_F_, Froude efficiency; *dv*, intracyclic variation of the swimmer’s velocity; *F*_r_, Froude number; *C*_Da_, active drag coefficient; *R*_e_, Reynolds number. 1, coaching level 1; 2, coaching level 2; 3, coaching level 3. ^∗^Significant differences (*p* < 0.05) between: 2 vs. 1, and 2 vs. 3.

Figure 2 depicts the selected variables, clustered by the coaching level held. The swimmers’ performance also improved based on the coaching level (1: 76.79 ± 11.27 s; 2: 75.06 ± 9.31 s; 3: 73.65 ± 8.43 s). The *C*_Da_ again denoted a significant variation, where significant differences were verified between groups 1 and 2 (*p* = 0.012, Δ = 28.30%, *d* = 0.92), and between groups 2 and 3 (*p* < 0.001, Δ = 43.24%, *d* = 0.93) (Table 1).

**FIGURE 3:**
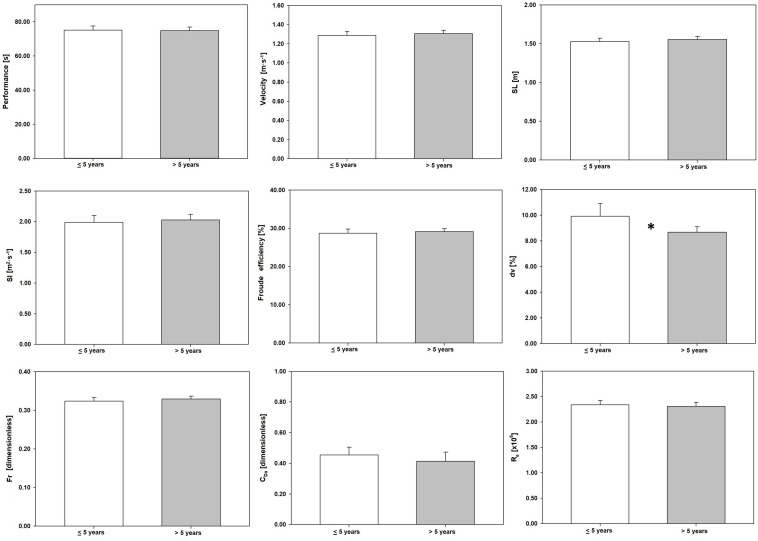
Performance and efficiency variables clustered by the coaches’ training experience. Performance, 100 m freestyle performance; velocity, swim velocity; SL, stroke length; SI, stroke index; η_F_, Froude efficiency; *dv*, intracyclic variation of the swimmer’s velocity; *F*_r_, Froude number; *C*_Da_, active drag coefficient; R_e_, Reynolds number. ≤5 years, 5 or less years of training experience; >5, more than 5 years of training experience. ^∗^Significant differences (*p* < 0.05).

Figure 3 presents the selected variables, clustered by coaching experience. Swimmers’ performance was better under a coach with longer careers (≤5 training experience: 75.44 ± 9.57 s vs. >5 training experience: 74.60 ± 9.54 s; 1.13%). The *dv* showed a significant difference between groups (*t* = 2.73, *p* = 0.007, Δ = 14.88%, *d* = 0.45) (Table 1).

In summary, based on bivariate analyses, swimmers benefited from being under coaches holding higher academic degrees, higher coaching levels, and longer careers. The follow-up question was how these factors could interact. To address such question, multivariate analysis (such as HLM modeling) was run.

Table 2 shows the data retrieved from the HLM model about the relationship between the coaches’ demographics and young swimmers’ performance. The model retained as main predictors all the coaches’ demographics [academic degree: estimate = -1.51, 95% confidence interval (CI) = -0.94 to -2.08, *p* = 0.014; coaching level: estimate = -0.92, 95% CI = -0.45 to -1.39, *p* = 0.031; coaching experience: estimate = -0.28, 95% CI = -0.14 to -0.42, *p* = 0.027] (Table 2). Moreover, the coaching experience also had a significant contribution to the swimmers’ *dv* (estimate = -0.12, 95% CI = -0.15 to -0.04, *p* = 0.029).

**TABLE 2:** Fixed effects of the final models computed with standard errors (SE) and 95% confidence intervals (95CI).

Fixed effect	Estimate (SE)	95CI	*p*-value
**Performance model**			
Intercept	79.46 (0.81)	77.87–81.05	<0.001
Academic degree	−1.51 (0.29)	−0.94 to −2.08	0.014
Coaching level	−0.92 (0.24)	−0.45 to −1.39	0.031
Coaching experience	−0.28 (0.07)	−0.14 to −0.42	0.027
***dv* model**			
Intercept	9.27 (0.12)	(9.03–9.51)	<0.001
Coaching experience	−0.12 (0.03)	(−0.15 to −0.04)	0.029

*dv, intracyclic variation of the swim velocity.*

## Discussion

The main purpose of this study was to understand the relationship between the coaches’ demographics and swimmers’ performance, swimming efficiency, and hydrodynamics. HLM suggested that an increase in all the coaches’ demographics had a positive and significant effect on young swimmers’ performance (being the academic degree the highest contributor). This contribution to the performance improvement is related to the enhancement of the variables related to swimming efficiency and hydrodynamics.

Our data showed that when swimmers were grouped by coaches’ demographics, an increase in each characteristic was related to an enhancement of the performance and all selected efficiency and hydrodynamics outcomes (Figures 1–3). Swimmers showing the best performances and better swimming efficiency and hydrodynamics were trained by the most experienced and qualified coaches. This suggests that these coaches may have a training perspective based on the efficiency and hydrodynamic enhancement, that is, quality of the swim technique, rather than in high volumes (quantity), leading to better performances (Nugent et al., 2017). Indeed, literature suggests that a long-term development approach, underpinned by high levels of technical efficiency and hydrodynamics, should be the bedrock of youth sports (Lang and Light, 2010; Morais et al., 2017).

Previous to the 2012 Youth Olympic Games, the excessive training loads in detriment of the development of technique got some attention based on the amount of mileage that young swimmers were being submitted to (Lang and Light, 2010). It was pointed out that 11- and 12-years-old swimmers may swim up to 32 miles a week (51.50 km), and 14 years about 40 miles a week (64.37 km) (Cassidy, 2008). This large mileage raises two major concerns: (1) it is not in tandem to what is suggested by literature, and (2) it could lead to a high ratio of dropouts. A study reported that 11- to 12-years-old swimmers should swim about 25 km per week (15.53 miles), and 13- to 14-years-old about 30 km (18.64 miles) (Chatard and Stewart, 2011). Thus, young swimmers were training 48.54% (11–12 years) and 46.60% (13–14 years) more than literature guidelines.

A study showed that during a 3-year period, time (i.e., training) did not have a significant effect on young swimmers’ performance enhancement (Morais et al., 2017). The variables that explained and predicted the performance were anthropometrics and stroke mechanics. The authors argued that growth and maturation and its interaction with biomechanics are the major drivers to performance enhancement. While growing, young swimmers will need to “relearn” and readapt their biomechanics to accommodate the shifts in anthropometric features. Coaches play a major role in such process (Morais et al., 2017). Moreover, the excessive amount of mileage can lead to a dropout phenomenon, because it removes the attraction of the sport, leading to a physical and mental burnout. Indeed, swimmers cite the emphasis on frequent, intense training as a main reason for dropping out (Cassidy, 2008). A study conducted with young swimmers noted that “demands,” “pressure,” and “dissatisfaction” were the dimensions or issues that better characterized the dropout and negatively predicted the intention of return (Monteiro et al., 2018). Moreover, such amount of training at early ages may inhibit musculoskeletal development and increase the likelihood of injuries. This will negatively impact performance and may present negative consequences in the swimmer’s growth development (Valovich et al., 2011).

All the variables related to the coach entered the final HLM as main predictors (Table 2). From those, the coaches’ academic degree had the highest contribution. An increase in the academic degree was related to a 1.51-s performance enhancement. These data showed that further academic studies led to an enhancement of the swimmers’ performance. It could be argued that the amount of knowledge about the performance determinant factors and a higher awareness on how to apply this knowledge and assessment procedures, that is, translating evidence into practice, facilitated swimmers’ improvement (Crowcroft et al., 2020). Additionally, coaching courses also allow coaches to gather substantial information not only on training volumes and intensity, but also on technical training (especially regarding young swimmers based on a long-term development). The manipulation of swimmers’ training volume (including aerobic and technical training) should promote physical adaptions through progressive overload (Carter et al., 2014). However, and at the same time, a key factor to a successful long-term development requires coaches to use relevant training load and specific training based on technical task-oriented drills (Chatard and Stewart, 2011; Lloyd et al., 2016). Thus, coaches who maintain an updated state-of-the-art about swimming training and long-term development may accurately prescribe and evaluate session’s intensity and orientation to avoid an inappropriate approach.

On the other hand, it was suggested that the barriers coaches face to access to sport science (for those who do not hold high academic degree, for instance) are the time required to find and read scientific journals and the lack of direct access to highly trained support staff, such as sport analysts, such as biomechanists (Reade et al., 2008). In the case of competitive swimming, young swimmers’ performance is highly driven by anthropometrics and biomechanics (variables related to technique, such as kinematics, hydrodynamics, and efficiency) (e.g., Morais et al., 2012; Barbosa et al., 2019). Thus, it seems that coaches who are aware and familiar with cutting-edge evidence about swimming determinants and have the skills to translate it into practice may have an edge in comparison to their peers.

The coaching experience showed a significant and inverse contribution to the swimmers’ *dv* (estimate = -0.12, *p* = 0.029) (Table 2). The *dv* is considered as an efficiency proxy (Barbosa et al., 2010). A larger intracyclic variation is related to more energy cost of transportation. So, it seems that coaching experience also provides coaches with knowledge to understand in which way they can help out their swimmers to improve the stroke mechanics, with the goal to minimize *dv* and therefore to excel.

Overall, it can be pointed out that despite all coaching features entered as significant predictors of young swimmers’ performance, the academic degree (i.e., level of scientific knowledge) was the highest contributor. Literature reported that young swimmers’ performance is based on interactions of several determinants and is deemed as a dynamic and highly complex system (Barbosa et al., 2010; Morais et al., 2017). Hence, a coach should be able to provide their athletes with training on key skills and abilities based in such determinant factors (Rezania and Gurney, 2014). In the past decade, research on age-group swimmers has been strongly focused on identifying and modeling the performance determinants (Morais et al., 2017; Barbosa et al., 2019) and analyzing young swimmers’ performance based on a talent identification approach (Yustres et al., 2020). However, little attention was given until now to coaches. They should facilitate the development of swimmers under them, designing programs that are underpinned by high-level and cutting-edge evidence. Coaches should be familiar with the most recent state of-the-art to help their swimmers to excel. Therefore, governing bodies such as national federations and regional associations should play an important role on (1) advising coaches to attend high-level coaching courses or preferably enrolling in higher academic degrees; (2) holding or sponsoring hands-on workshops based on cutting-edge evidence and who translate it to practice; and (3) supporting coaches by facilitating access to new trends and novel and impactful knowledge in youth swimming research.

As main limitations, the lack of information on training programs designed by the recruited coaches (e.g., the disclosure of the mileage covered by swimmers) and that these estimations are only for the 100 m freestyle event can be considered. The following can be suggested: (1) a cluster analysis in the future to understand if the fastest swimmers were characterized by higher technical/efficiency parameters and under the coaches with the highest expertise and experience and (2) the assessment of psychological variables to learn the relationship between the coaches’ demographics (academic degree, coaching level, training experience) in the applied training content and the swimmers’ psychological profile and performance.

## Conclusion

The coaches’ demographics (i.e., academic degree, coaching level, and coaching experience) have a significant and positive relationship with young swimmers’ performance and swimming efficiency. This shows that coaches familiar with an up-to-date knowledge can design a more effective development program with a larger likelihood of better performances by swimmers under them. Among those, the academic degree showed the largest contribution. The faster and more efficient swimmers were under coaches holding high academic degrees. Therefore, further and long-term learning over the coaching career, regardless of the path selected (higher academic degree and/or high-level coaching courses), concurrent to coaching experience is a must for young swimmers to deliver good performances.

## Data Availability Statement

All datasets generated for this study are included in the article/supplementary material, further inquiries can be directed to the corresponding author.

## Ethics Statement

The studies involving human participants were reviewed and approved by the University of Beira Interior Review Board. Written informed consent to participate in this study was provided by the participants’ legal guardian/next of kin.

## Author Contributions

DM, TB, and JM conceived and designed the experiments, edited and reviewed. DM, PF, and JM performed the experiments. VL, TB, and JM analyzed the data. DM, TB, VL, PF, AT, and JM wrote the original draft. All authors contributed to the article and approved the submitted version.

## Conflict of Interest

The authors declare that the research was conducted in the absence of any commercial or financial relationships that could be construed as a potential conflict of interest.
